# Astrocytes and the Warning Signs of Intracerebral Hemorrhagic Stroke

**DOI:** 10.1155/2018/7301623

**Published:** 2018-02-04

**Authors:** Annalisa Scimemi

**Affiliations:** SUNY Albany, Department of Biology, 1400 Washington Avenue, Albany, NY 12222, USA

## Abstract

Two decades into the two thousands, intracerebral hemorrhagic stroke (ICH) continues to reap lives across the globe. In the US, nearly 12,000 people suffer from ICH every year. Half of them survive, but many are left with permanent physical and cognitive disabilities, the severity of which depends on the location and broadness of the brain region affected by the hemorrhage. The ongoing efforts to identify risk factors for hemorrhagic stroke have been instrumental for the development of new medical practices to prevent, aid the recovery and reduce the risk of recurring ICH. Recent efforts approach the study of ICH from a different angle, providing information on how we can limit brain damage by manipulating astrocyte receptors. These results provide a novel understanding of how astrocytes contribute to brain injury and recovery from small ICH. Here, we discuss current knowledge on the risk factors and molecular pathology of ICH and the functional properties of astrocytes and their role in ICH. Last, we discuss candidate astrocyte receptors that may prove to be valuable therapeutic targets to treat ICH. Together, these findings provide basic and clinical scientists useful information for the future development of strategies to improve the detection of small ICH, limit brain damage, and prevent the onset of more severe episodes of brain hemorrhage.

## 1. Introduction

Preserving the function of the brain throughout the course of a lifetime is a challenging task that requires the coordinated efforts of healthy neurons, glial cells, and blood vessels. The physiological equilibrium created by these cells breaks during brain injury, as in the case of ICH. Although glial reactivity has been well documented in pathological studies of ICH, the structural and functional changes associated with it were initially interpreted as representing a downstream effect or a “reactive” response to neuronal damage [1]. Recent evidence challenges this interpretation suggesting that glial cells are “active” contributors to brain damage, meaning that glial pathology is part of the disease progression.

Blood extravasation during ICH damages neurons, glial cells, and blood vessels. Therefore, recovering from ICH requires restoring the function of all these cells and structures. Traditionally, pharmacological approaches to treat ICH have targeted molecules implicated with the blood coagulation cascades and/or molecules associated with neurons, leaving out glial cells from the scene of potentially valuable therapeutic targets. To date, there is no drug currently available on the market that specifically targets glial cells to treat brain damage caused by ICH [1].

Astrocytes are glial cells with fine processes closely associated with synapses, enriched with a high density of neurotransmitter transporters [2, 3]. Through the activity of these transporters, astrocytes shape the time course of synaptic transmission among neurons. Through the activity of K^+^ channels (e.g., Kir4.1), astrocytes maintain the extracellular K^+^ concentration at levels that are compatible with life [4, 5]. Through their aquaporin-rich endfeet at the cerebral capillaries, astrocytes control the bidirectional movement of water across the cell membrane [5]. Last, astrocytes secrete proinflammatory (IL-6 and IL-1*β*) and anti-inflammatory cytokines (IL-10) and chemokines (CCL2, CXCL1, CXCL10, and CXCL12) through which they control microglia differentiation and macrophage activation [6–10]. Because of their involvement in such a wide variety of molecular processes, it is interesting to exploit the potential that astrocytes may have as therapeutic targets to treat ICH.

In this review, we first describe ICH and the molecular events that take place during blood extravasation from the brain vasculature. We summarize the current knowledge on the risk factors for ICH and discuss how astrocytes could contribute to limit brain damage caused by ICH.

## 2. Intracerebral Hemorrhagic Stroke: What Is It?

ICH is a medical emergency with potentially devastating effects, caused by the sudden rupture of one or more blood vessels in the brain. In most cases, the bursting blood vessels are small-penetrating arteries or arterioles in the deep subcortical regions, cerebellum, and brainstem. Blood leakage triggers cell death in the surrounding neuropil, ultimately leading to impaired cognitive abilities and dementia [11–15].

ICH induces primary and secondary damage to the brain. The primary damage, known as the “mass effect,” is due to the mechanical compression of the brain caused by local blood accumulation. The secondary damage is due to (1) cytotoxicity of the blood; (2) excitotoxicity, due to the release of excitatory amino acids like glutamate from injured neurons; (3) spreading depression, a slow and short-lived depolarization wave that propagates through the brain; (4) hypermetabolism, a state of increased energy expenditure in response to injury; and (5) oxidative stress and inflammation [16].

The infiltration of blood components in the brain is important for triggering the release of inflammatory factors that contribute to the activation of macrophages and microglial cells [17]. The enhanced local production of pro-inflammatory cytokines contributes to disrupt the blood-brain barrier and leads to the development of perihematomal edema, which in turn amplifies the mass effect. The activation of macrophages and microglial cells exerts also a neuroprotective effect because it promotes the removal of damaged tissues, which is an essential step towards recovery. Through the activity of macrophages and microglial cells, the brain reacts immediately to the smallest ICH and in many cases succeeds in its attempts to preserve neuronal function. Despite this, ICH remains a harmful event even when it is small and confined, because primary and secondary damage can disrupt the function of complex neural networks [18]. These effects are often long lasting and ultimately lead to cognitive dysfunction, emotional lability, fatigue, depression, and suicidality [19–22].

The disruptive and neuroprotective nature of many of the molecular events associated with ICH highlights the complexity of this pathology and the need to understand ICH in its finest molecular details, starting from the risk factors, in an attempt to improve its detection, prevention, and treatment.

## 3. The Vulnerable Brain: Risk Factors for Intracranial Hemorrhagic Stroke

A small proportion of ICHs is due to vascular malformations or tumors, but the vast majority is due to various and only partly known risk factors. Some are associated with an individual's lifestyle and are therefore modifiable. For example, poorly controlled hypertension, smoking, and regular heavy alcohol consumption are known, modifiable risk factors for ICH in young and middle-aged people [23]. ICH can also arise as a secondary effect of other pathologies including cerebral amyloid angiopathy and Alzheimer's disease. Risk factors like aging are nonmodifiable. During aging, there is an increased deposition of amyloid proteins in cortical arterial blood vessels, which is a predisposing factor to ICH due to its ability to degrade the elasticity of the blood vessels wall, rendering them more susceptible to rupture [24].

Recent advances in high-throughput genotyping technologies, big data analysis, genome-wide association studies, and the creation of large international consortia [25] have led to the identification of genetic risk factors that vary depending on the brain region affected by ICH [26, 27]. According to these studies, the *ε2* and *ε 4* alleles of the apolipoprotein E (APOE) are independent genetic risk factors for cortical ICH [28]. APOE is essential for lipoprotein catabolism, glucose use by neurons and glial cells, and synapse maintenance and plasticity. The *ε2* and *ε4* alleles of APOE are implicated with the pathogenesis of cerebral amyloid angiopathy [29–39]. The larger is their allele copy number, the greater the severity of the ICH (i.e., the hemorrhage size and growth [40]). Risk factors for deep, subcortical ICH include variants of the genes *PMF1*, *SLC25A44*, and *SEMA4A* which encode the polyamine-modulated factor 1, a mitochondrial transmembrane transporter and a member of the semaphorin family implicated with axon guidance and immune response, respectively [39, 41].

These results highlight the heterogeneity in the risk factors for different types of ICH and the need for a better understanding of the molecular mechanisms underlying ICH to generate new hypotheses for its treatment. Because brain damage induced by ICH affects all cell types surrounding ruptured blood vessels and because astrocytes are abundant cells in the brain, it is interesting to consider how astrocytes function, respond to ICH, and contribute to the recovery from brain injury.

## 4. Astrocytes: Bidirectional Control of Blood Flow and Neuronal Function

**Figure 1:**
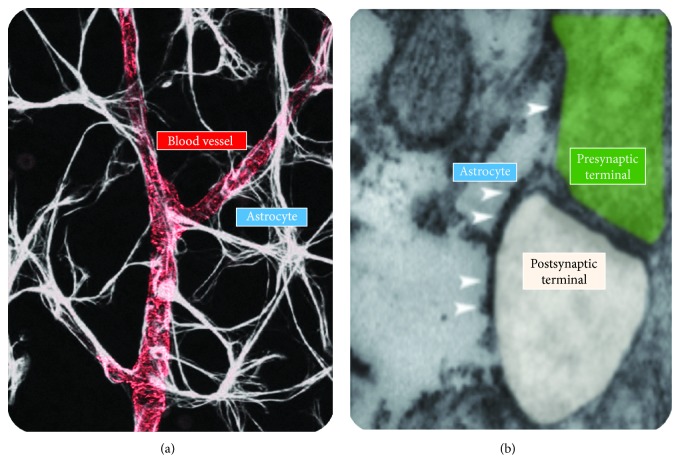
Astrocytic interactions with blood vessels and synapses. (a) GFAP labeling of astrocytes (*white*) making contact with blood capillaries, visualized using antibodies directed against the smooth muscle-specific *α*-actin (ASMA) (*red*). Modified with permissions from [126]. (b) Transmitted electron micrograph of an excitatory synapse in the hippocampal area CA3, contacted by an astrocytic process. Arrowheads show PAR1 localization along the astrocytic membrane. Modified with permissions from [80].

Astrocytes are intriguing in the context of ICH because their fine processes are in tight contact with both blood vessels and synapses (Figure 1). This means that astrocytes are capable of coupling changes in blood flow to changes in neuronal function and vice versa.

The astrocytes' perivascular endfeet ensheathe blood vessels tightly through transmembrane-anchoring proteins including AQP4, the potassium channel Kir4.1, and their adaptor proteins syntrophin, dystrophin, and dystrobrevin [42–45]. Through these processes, astrocytes regulate the transport of water and other molecules from the lumen of blood vessels to the surrounding brain tissue [42, 46]. This close interaction is important for the formation and maintenance of the blood-brain barrier and forms the basis for coupling vasodilation/constriction and neural activity [5, 47, 48].

The astrocytes' perisynaptic endfeet are enriched with glutamate transporters, which are responsible for limiting the lifetime of glutamate in the extracellular space and the time course of glutamate receptor activation [2, 49]. Glutamate transporters bind glutamate rapidly as it diffuses from the synaptic cleft towards the extracellular space. The glutamate binding rate of excitatory amino acid transporters (EAATs *K*_on_: 5 × 10^6^ M^−^^1^·s^−^^1^ [50]) is similar to the glutamate-binding rate of GluA and GluN receptors (GluA *K*_on_: 28.4 × 10^6^ M^−1^·s^−1^ [51]; GluN *K*_on_: 5 × 10^6^ M^−1^·s^−1^ [50, 52]). Once bound to EAATs, glutamate has a 50% chance of being translocated to the cell cytosol because the translocation efficiency of glutamate transporters, representing the proportion of glutamate molecules initially bound to the transporter that are eventually moved across the membrane, is only 50% [53]. The remaining 50% of glutamate molecules are released back in the extracellular space, a phenomenon commonly referred to as “buffering” [50, 53, 54]. What happens to the glutamate buffered by EAATs and released back in the extracellular space: does it bind to other EAATs or to GluA/N receptors? If we consider glutamate binding to a substrate as a simple first-order chemical reaction, the likelihood with which glutamate unbinding from EAATs binds to one substrate or another depends on the glutamate-binding rate and the concentration of the substrate. Given the relatively similar binding rate of glutamate to receptors and EAATs, whether buffered glutamate binds to one or the other depends on the local density of receptors and EAATs in the extra-synaptic environment. The density of expression of extra-synaptic glutamate receptors is 1000 times lower than that of EAATs in astrocytes (GluA/N density: ~10 *μ*m^−2^ [55–57]; EAAT density: ~10,800 *μ*m^−2^ [49]). This means that it is 1000 times more likely for glutamate unbinding from EAATs to bind to other EAATs instead of binding to glutamate receptors. Therefore, glutamate buffering can prolong the lifetime of glutamate in the extracellular space without necessarily prolonging the time course of glutamate receptors activation [58]. Once bound to EAATs for the second time, glutamate has again a 50% chance of being removed from the extracellular space and of being released back in the extracellular space. This type of iterative process leads to a progressive dilution of glutamate in the extracellular space, until glutamate reaches its final, low nanomolar, steady-state ambient concentration [59, 60].

**Figure 2:**
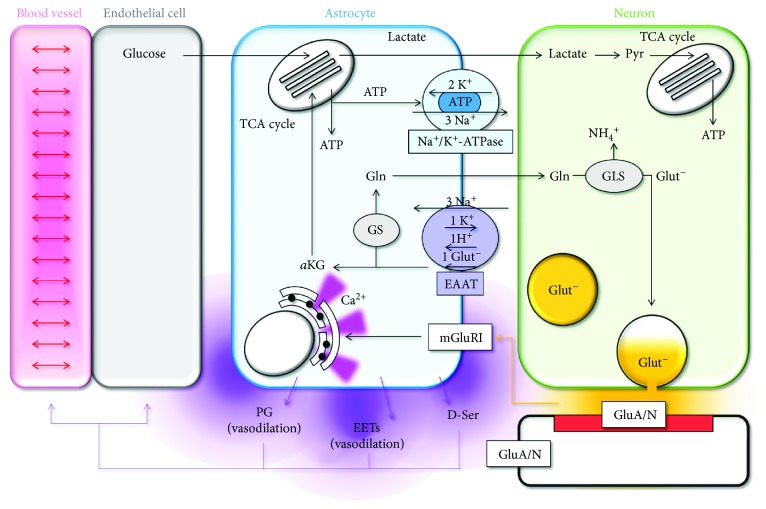
Astrocytic molecular pathways mediating neurovascular coupling. The figure summarizes the molecular pathways that couple neuronal activity at excitatory synapses with glutamate uptake in astrocytes and vasodilation in blood vessels. Conversely, glucose metabolism in endothelial cells supports excitatory synaptic transmission in neurons by fueling energy production in astrocytes. *α*KG: *α*-ketoglutarate; D-Ser: D-Serine; GS: glutamine synthase; EAAT: excitatory amino acid transporter; EETs: epoxyeicosatrienoic acids; Glut^−^: glutamate; Gln: glutamine; GLS: glutaminase; mGluRI: group I metabotropic glutamate receptors; PG: prostaglandins; Pyr: pyruvate; TCA: tricarboxylic acid cycle.

Glutamate molecules taken up by astrocytes set off for a complex journey (Figure 2). In the astrocyte cytoplasm, the enzyme glutamine synthetase (GS) converts part of them into glutamine. Glutamine is transferred back to neurons to be used as a substrate for the enzyme glutaminase (GLS), which removes an amide group from glutamine to produce glutamate and ammonia [61] (Figure 2). The amount of glutamate returned to neurons is not a fixed proportion of the amount of glutamate taken up by astrocytes but depends on both the amount of glutamate returned to neurons through transporters and the amount synthesized de novo from glutamine [62]. Glutamate molecules that do not enter the glutamate-glutamine cycle are converted into *α*-ketoglutarate (*α*KG) which is used as a substrate for the tricarboxylic acid (TCA) cycle in astrocytes to cover, at least in part, the energy demand of the glutamate uptake process [63] (Figure 2). The rest of the energy costs of the glutamate uptake process are covered by harnessing the electrochemical gradient for Na^+^, K^+^, and H^+^ ions, which are moved across the membrane by glutamate transporters in fixed ratio with glutamate (3 Na^+^, 1 K^+^, 1 H^+^, and 1 Glut^−^ [64, 65]; Figure 2). The energy required to establish these ionic gradients comes mostly from primary active transporters that establish the electrochemical gradient for Na^+^ and K^+^ ions, like the Na^+^/K^+^-ATPase (Figure 2). The production of ATP necessary to support the Na^+^/K^+^-ATPase takes place in the mitochondria and requires, in part, glucose consumption, which fuels energy back to the neurons through the production of lactate [66] (Figure 2). The source of glucose for astrocytes is the endothelial cells around the blood vessels. Therefore, changes in glucose metabolism in endothelial cells, which are affected by changes in the O_2_/CO_2_ balance in the bloodstream, shape the ability of astrocytes to take up glutamate from the extracellular space. This regulation of glutamate uptake by the O_2_/CO_2_ balance in the bloodstream carries important functional implications because of its ability to control excitatory synaptic transmission.

In turn, changes in neuronal activity affect blood flow by altering astrocytic function. Accordingly, increased synaptic activity induces an increase in blood flow through signaling mechanisms that couple glutamate release to activation of group I metabotropic glutamate receptors (mGluRI) and increased intracellular Ca^2+^ concentration in astrocytes. This rise in intracellular Ca^2+^ concentration triggers the release of signaling molecules like prostaglandins (PG), epoxyeicosatrienoic acids (EETs), and D-Ser that act on arterioles and pericytes around the capillaries to induce vasodilation/constriction [67–70] (Figure 2). Through these mechanisms, glutamate transporters communicate to the astrocytes the need to adjust blood flow to the level of synaptic activity at a particular time, in a particular region of the brain [71].

Therefore, astrocytes are in a key position to mediate neurovascular coupling as they can adjust the level of synaptic strength in response to changes in blood flow and they can modify blood flow in response to ongoing synaptic activity. The ability to exert this type of bidirectional control puts astrocytes in a unique position in the context of brain injury induced by ICH.

## 5. The Role of Astrocytes in ICH

During ICH, a number of molecules that typically reside in the bloodstream quickly invade the surrounding brain tissue, damaging not only neurons but also astrocytes and blood vessels. Astrocytes occupy a substantial portion of the brain tissue, and a complete impairment of their functions is incompatible with life. In ICH, astrocytes undergo important structural and functional modifications that can either be neuroprotective or detrimental for neuronal function and therefore need to be understood in further detail [72].

**Figure 3:**
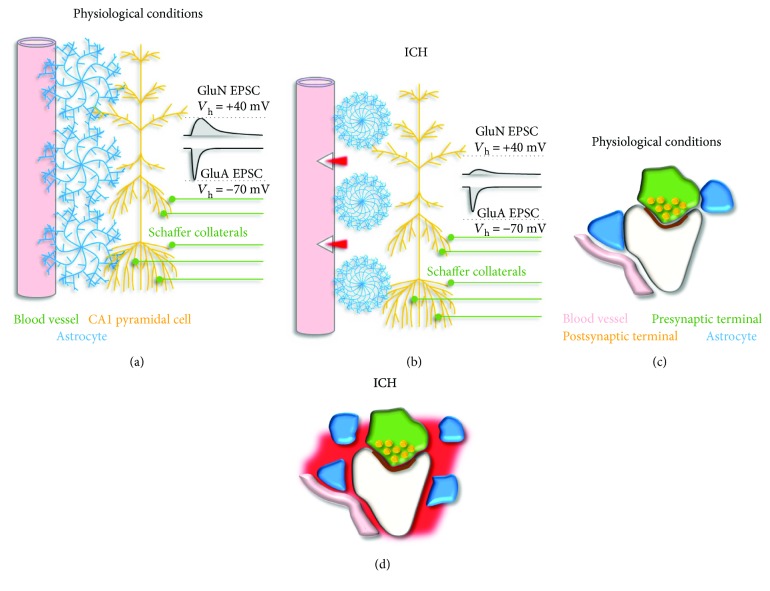
The coupling of astrocytes with blood vessels and synapses changes during ICH. (a) Schematic representation of the organization of blood vessels (*pink*), astrocytes (*blue*), and pyramidal cells in the hippocampal area CA1 (*yellow*) in physiological conditions. Astrocytes closely interact with both blood vessels and neurons. CA1 pyramidal cells receive excitatory afferents from area CA3 via the Schaffer collateral pathway (*green*). GluA and GluN receptors are expressed postsynaptically at Schaffer collateral synapses. Current responses mediated by GluA and GluN receptors can be isolated using pharmacological approaches and whole-cell patch-clamp recordings in voltage-clamp mode holding pyramidal cells at −70 mV or +40 mV, respectively (*black*). (b) Schematic representation of the structural reorganization of astrocytes in response to ICH and PAR1 activation. Astrocytic processes shrink, proliferate, and migrate further away from excitatory synapses. These changes cause faster glutamate clearance and weaken GluA- and GluN-mediated synaptic transmission [87]. (c) Schematic representation of the synaptic and perisynaptic environment in physiological conditions. (d) Schematic representation of the synaptic and perisynaptic environment in ICH. The remodeling of the neuropil induced by ICH is as described in (b).

One of the consequences that blood extravasation has on astrocytes pass through the activation of coagulation cascades that cleave the precursor protein prothrombin to generate the serine protease thrombin [73]. Thrombin causes brain damage because it induces perihematomal edema formation [74–76] and leads to the activation of members of the serine protease-activated G-protein-coupled receptor (PAR) family [77–79]. One of these receptors, called PAR1, is predominantly localized to the perisynaptic astrocytic endfeet [80] and shows continued activation following ICH [81]. Thrombin cleaves the extracellular N-terminal domain of PAR1. The newly exposed N-terminal domain acts as the tethered ligand for PAR1, which in turn triggers activation of G_i/o_, G_q/11_, and G_12/13_ signaling pathways [82–84]. The fact that PAR1 activation contributes to brain damage during ICH is supported by evidence that mice lacking PAR1 have reduced brain infarct volume during focal ischemia [85, 86]. However, recent evidence indicates that PAR1 activation also induces rapid remodeling of astrocytic processes adjacent to glutamatergic synapses [87]. This remodeling includes (1) shrinkage and flattening [87, 88], (2) proliferation [87, 89], and (3) migration of astrocytic processes away from excitatory glutamatergic synapses [87] (Figure 3). These structural changes occur rapidly (within 20–30 min) and locally (within a radius of few hundreds of nanometers from excitatory synapses). Despite its local nature, this remodeling of the perisynaptic environment carries important functional implications, because it leads to an increase in the local glutamate uptake capacity. The experimental data suggests that the postsynaptic response to sparse excitatory inputs is weaker in response to PAR1 activation, as fewer GluA receptors open in response to a single glutamate release event [87]. At the same time, fewer GluA receptors enter the desensitized state. Therefore, postsynaptic responses to high-frequency stimuli summate more efficiently during PAR1 activation. In other words, PAR1 activation converts excitatory synapses into high-pass filter devices, well tailored to relay information associated with high-frequency—not sparse—neuronal activity [87]. For reasons that are not clear, these findings differ from initial reports suggesting that PAR1 activation does not change GluA-mediated synaptic transmission in the rat hippocampus [90, 91].

The effect of PAR1 activation on GluN receptors is important for the role that these receptors play in the induction of long-term plasticity [92, 93]. Long-term potentiation (LTP) and depression (LTD) are widespread phenomena across different brain regions, extensively studied at Schaffer collateral synapses in the hippocampal area CA1, where they are thought to be the substrates of memory formation [94]. At Schaffer collateral synapses, the Mg^2+^-block of GluN receptors is incomplete at rest [95]. Because the driving force for Ca^2+^ is large, a significant amount of Ca^2+^ enters postsynaptic terminals via GluN receptors in response to presynaptic stimulation and glutamate release. The magnitude, temporal profile, and spatial spread of the evoked rise in intracellular Ca^2+^ concentration determine the direction of the evoked changes in synaptic strength. The reduced GluN activation associated with PAR1-induced remodeling of astrocytic processes leads to impairment of long-term plasticity [87]. This suggests that AR1 activation may be implicated with cognitive impairment caused by ICH, through mechanisms involving structural plasticity of the extra-synaptic environment. Do these effects contribute to brain damage during ICH or are they neuroprotective? The reduced activation of GluA/N receptors may seem detrimental, as it weakens the strength of excitatory synaptic transmission. The high-pass filtering effect may also be disruptive as it promotes high-frequency activity and seizure propagation in the brain. On the other hand, speeding glutamate clearance could prevent glutamate-induced excitotoxicity, which would serve a neuroprotective role. In this case, PAR1 receptors would act as an imperfect safety mechanism through which the brain prevents excitotoxicity at the expenses of weaker synaptic transmission and higher risks of seizure propagation [87].

Other works have identified specific molecular mechanisms by which PAR1 affects astrocytic function. For example, PAR1 activates the p44/42 mitogen-activated protein kinase ERK1/2 in cultured astrocytes [96, 97]. In addition, the submicromolar increase in intracellular Ca^2+^ concentration evoked by PAR1 activation directly activates the bestrophin-1 channels (Best1) [98]. In the rat hippocampus, this has been suggested to cause increased [99–101], rather than decreased [87], GluN activation. These findings are consistent with data obtained in the nucleus of the solitary tract, where PAR1 agonists also lead to increased GluN activation [102]. They are also consistent with reduced GluN-dependent LTP and learning and memory deficits in PAR1^−/−^ mice [103, 104], but they are odds with data obtained in the mouse hippocampus [87] and therefore require further investigation.

Whether PAR1 activation modulates GABAergic transmission remains unclear. In the cerebellum, Best1 activation induces GABA release from astrocytes, leading to increased tonic inhibition [99]. However, the jury is still out on whether plasmin-induced activation of PAR1 affects phasic GABAergic transmission in the hippocampus, given that there is experimental evidence in favor [91] and against it [90].

The existence of discrepancies among the experimental findings of different labs inevitably calls for additional experiments and further validation. It is likely that PAR1, being coupled to multiple signaling pathways, might exert a plethora of effects that are better understood by interfering specifically with one signaling pathway or another.

## 6. Can Astrocytes Be Valuable Targets to Develop Future Treatments for Intracranial Hemorrhagic Stroke?

If PAR1 activation somehow contributes to mediate the unwanted consequences of blood extravasation on cognition, it is tempting to think that its blockade may also contribute to limit brain damage caused by ICH. In humans, astrocyte proliferation in the brain is detected adjacent to both ischemic and hemorrhagic lesions [105]. A number of peptide-based agents, small molecules, and proteases have been identified over the years to block PAR1, and some of them have progressed to clinical trials [106]. One of the main challenges when comparing the effects of these compounds in animal models and in humans is that the cellular expression of PAR1 varies across species and is not limited to the central nervous system. For example, humans, monkeys, and guinea pigs express PAR1 in platelets. Nonprimate species (e.g., rodents, dogs, and rabbits) do not express PAR1 in platelets but do so in other peripheral tissues [107]. Among all PAR1 antagonists, the one that has been studied most extensively is vorapaxar (SCH50348, Zontivity™).

Vorapaxar is an irreversible PAR1 inhibitor that binds to PAR1's primary binding site. Vorapaxar was not developed to deliberately target PAR1 receptors in astrocytes but to act on PAR1 receptors in platelets, in an attempt to block platelet activation and decrease the risk of cardiovascular events. It was the first PAR1 antagonist approved for use in clinical studies to reduce thrombotic events in patients with a history of myocardial infarction and peripheral arterial disease without history of stroke or transient ischemic attack. Vorapaxar is not administered to patients that already suffered a transient ischemic attack or stroke, due to its effect on increasing bleeding risk. In two large phase III clinical trials (TRACER [108] and TRA 2°P-TIMI 50 [109]), vorapaxar administered as an add-on therapy on top of aspirin and clopidrogel led to reduced incidence of ischemic events but an increased incidence of intracranial hemorrhage. Because of all these undesirable effects, there are concerns on the usefulness of PAR1 blockade to prevent or treat brain damage in ICH, further emphasizing the need for new studies on the molecular properties of PAR1, function, and modulation, in and out of the brain.

Besides PAR1, astrocytes possess a number of other molecules that can serve as molecular targets during ICH. For example connexin 43, a major connexin subtype in astrocytes, changes its expression in ischemia and stroke [110]. A2A adenosine receptors in astrocytes are involved in neuroinflammatory and neuromodulatory processes an in the regulation of glutamate homeostasis, all implicated with ischemic brain injury [111]. Meteorin, a 291 amino acid peptide secreted by astrocytes, acts on endothelial cells and regulates angiogenesis [112]. Another compound produced by astrocytes, octadecaneuropeptide (ODN), prevents oxidative stress-induced apoptosis. Its ability to reduce neuronal damage makes it a particularly valuable candidate in ICH [113, 114].

The value of considering molecular targets in astrocytes comes from evidence that neurons are not viable without astrocytes [115], and astrocyte viability is maintained for longer than neurons in animal models of stroke [116–118]. For this reason, targeting surviving astrocytes offers an invaluable opportunity to restore the function of neurons and blood vessels in the damaged brain [72]. Accordingly, gene delivery studies show that promoting astrocyte survival protects against stroke [119]. The path to discovering new pharmacological tools to target molecules in astrocytes requires, first and foremost, an appreciation of the pivotal function of these cells to ensure functioning of the healthy brain. It requires extensive research and molecular screening, as it happens when searching for any new target molecule in other cell types [1]. Once the candidate target molecule is found, the expectation is that it is druggable and that the drugs that act on it are specific and safe [1]. Druggable means that it has to be targeted by a chemical compound with high affinity. Traditionally, G-protein-coupled receptors like PAR1 are considered to be the best druggable targets [120]. However, recent technical advances open new opportunities that allow modulating protein-protein interactions [121] and facilitating antibody penetration through the blood-brain barrier [122–124]. Specificity, one of the most desirable features of a drug, refers to its ability to act only on a given target molecule. This can be challenging to accomplish because the predicted chemical and actual biological specificity of a compound may differ substantially [125]. Safety, commonly assessed by testing a compound on animals, poses further challenges because some molecular targets like PAR1 are differently expressed in animal models and humans, in tissues other than the brain and across different brain regions. While the quest for the best therapeutic strategy for ICH continues, it is important for basic scientists to continue to broaden our knowledge of the molecular pathways that allow astrocytes to shape the functional properties of the brain.

## 7. Conclusions

Astrocytes are in a strategic position to shape neuronal function and blood flow. For this reason, they provide a broad range of opportunities for therapeutic intervention aimed at restoring neuronal function and blood flow in brain regions affected by ICH. The potential of astrocytes as therapeutic targets to prevent or treat ICH has not been fully exploited. New experimental evidence indicates that small changes in the structure of astrocytic processes at excitatory synapses can change profoundly the strength of synaptic transmission in the brain. This, in turn, can have profound implications for regulating cognitive skills. Therefore, while searching for new molecular targets to treat ICH, it is important to keep an open mind on molecular targets in nonneuronal cells. Astrocytes, for once, may be in a privileged position to help.
